# Assessment and Referral of Patients With Short Stature at Primary Care Buraidah, Saudi Arabia

**DOI:** 10.7759/cureus.86197

**Published:** 2025-06-17

**Authors:** Futun Almutairi, Unaib Rabbani

**Affiliations:** 1 Family Medicine Academy, Qassim Health Cluster, Buraydah, SAU

**Keywords:** growth hormone, kingdom of saudi arabia (ksa), management, primary care, short stature

## Abstract

Background and objectives: Short stature (SS) causes a significant social and mental burden on patients and their families. Primary healthcare (PHC) physicians (Family Medicine) are crucial in securing the timely evaluation and referral of children with SS. This research aimed to assess the management and referral of short-stature patients by PHC physicians about SS in Buraidah, Saudi Arabia.

Methods: A cross-sectional study was conducted among PHC physicians in Buraidah from November 2024 to February 2025. A validated closed-ended questionnaire was used to collect data online. Descriptive and inferential analyses were carried out to assess knowledge levels, attitudes, practices, and factors influencing their knowledge of SS. Data was analyzed using the Statistical Package for Social Sciences (SPSS).

Results: A total of 143 PHC physicians participated in the study. Mean knowledge score (standard deviation) was 7.0 (±2.47) out of 13. About 99 (69%) would refer a patient once the height falls below the 5th percentile. More than half, 79 (55%), considered growth hormone as an option for the treatment of SS. Seventy-two percent used local growth charts. The most common tests requested before referral of SS patients were complete blood count (CBC) 110 (77%) and thyroid function test 109 (76%). The majority (79% to 98.6%) of participant indicated their learning needs on various aspects of SS. There was no significant difference in the knowledge score with respect to socio-demographic and professional characteristics except for nationality.

Conclusion: Knowledge level of PHC physicians in Buraidah is moderate (68%), with the majority indicating their learning needs on this topic. The study’s findings call for focused educational initiatives and policy enhancements to advance healthcare delivery for children with SS in Buraidah, Saudi Arabia.

## Introduction

Short stature (SS) is among the major health problems faced by the pediatric group globally [1]. Furthermore, SS is the most common indication for referral to pediatric endocrinologists. However, most of the referred children have normal growth variants, such as familial short stature (FSS), and constitutional delay of growth and puberty (CDGP) [2,3]. Additionally, SS is frequently identified at a late age, resulting in the late initiation of the treatment [1,4]. Moreover, the guidelines and clinical practice to identify children with SS differ, leading to variations in prevalence across countries worldwide [2]. In this regard, a cross-sectional study in a south Indian district reported SS with an overall prevalence of 2.86% (n= 448) among 15644 school children [5]. Similarly, the prevalence was reported to be 3.26% among children and adolescents in Shanghai, China, and 3.16% among primary and middle school students in Anhui, China [6]. In the UK, a prevalence of 1.3% (n=180) was observed among 1400 children at school entry [2]. Interestingly, a 2004-2005 study (n=19,372 children and adolescents) in Saudi Arabia reported a relatively higher prevalence of SS for children and adolescents, with 11.3% and 1.8% in males, as well as 10.5% and 1.2% in females, respectively [1]. Similarly, out of 110 Saudi children with SS, the male-to-female ratio was 1.3:1. Genetic SS was the most common in 51.8% (n= 57) of the cases, while idiopathic growth hormone (GH) deficiency was the most prevalent in 21.8% (n= 7) of the remaining 53 patients [7]. Furthermore, in a study involving 9018 Saudi children and adolescents, the SS prevalence was found to be higher in the Southwestern = (%)compared to the Northern or Central (%) regions of Saudi Arabia (P <0.0001), with males accounting for 51% of the cases [8].

Early diagnosis of SS and the subsequent initiation of treatment are imperative for a better prognosis, especially in pediatric candidates for GH treatment [2,4]. Thus, improving the referral process in a timely and efficient manner is essential to reduce or even prevent unneeded referrals, along with the subsequent healthcare burden and anxiety for families, while increasing the number of early and accurate referrals on the other hand [4,9]. Child healthcare providers, including family medicine physicians, pediatricians, pediatric endocrinologists, and pediatric subspecialists, play a vital role in the assessment and management of children with growth disorders, particularly SS [4].

The diagnosis of SS usually includes anthropometric measurements (i.e., height vertex, and body weight), and medical tests (i.e., GH provocation test, IGF-1 levels, complete blood count, and bone age X-ray) [10]. Therefore, the child healthcare provider must have deep expertise and effective training to gather a detailed medical history and conduct an accurate physical examination of the children of interest, in order to facilitate an effective diagnostic workup [2]. In this regard, a US-based study (n= 1268 pediatricians), found that the referral decisions were influenced by patient indicators (such as height and growth pattern), family concerns, and physician attitudes, particularly in children with less severe growth retardation (P <0.001). Additionally, the physicians’ characteristics (such as being female, shorter, or older) were associated with increased referral rates (P <0.03-0.001) [11]. Similarly, Silvers et. al. reported that recommendations to initiate GH treatment were influenced by family preferences and physician attitudes as much as the relevant guidelines (P <0.001) [12]. Likewise, in their nationwide survey on pediatric endocrinologists, Hardin et. al. concluded that the participants (n=182) indicated poor target height and growth velocity, along with low IGF-1 levels, as factors for prescribing GH therapy for patients with GH deficiency, particularly in idiopathic short stature (ISS). Furthermore, 62% of participants considered the cost of GH in their treatment decision, while 55% were influenced by concerns about the future unknown side effects of GH treatment, and 37% were influenced by family persistence. Although 82% of the participants were already using GH treatment for ISS, 69% indicated GH treatment for ISS. Additionally, 78% of them perceived SS as a disability [13].

In a meeting of nine pediatric endocrinology experts from the Gulf Cooperation Council (GCC) and three peers from Europe, seven of the GCC experts reported their adherence to international guidelines rather than national guidelines for GH treatment in patients with GH deficiency, particularly children with SS. Some experts expressed major concerns regarding efficacy (5/12) and cost (4/12). The GCC experts considered height velocity (n=7), IGF-1 levels (n=6), and height SD score (n=5) as essential investigations [14]. Moreover, Kaplan et al. conducted a multinational cross-sectional study on 450 participants across the GCC countries, with the majority (37.8%) being from Saudi Arabia [4]. The study reported that 82.8% of the participants correctly identified the term SS. However, only 24% of the participants consistently used a wall-mounted stadiometer. Regardless of GH deficiency or ISS, approximately 50% of the participants considered referring children with SS due to other clinical conditions. Forty-one percent of the participants considered referring children with SS at a height below the 5th percentile. While 57% of the participants chose GH as a treatment option for those children, only 60% of them considered it safe [4]. Indeed, the low sample size of this study is a major block for the inference of these results on a wide scale across the GCC countries; more large-scale studies are needed, especially in Saudi Arabia and its different regions. Moreover, in the survey study by Al-Herbish et al., all 52 Saudi physicians indicated GH treatment for GH deficiency. Although 17% and 19% of the participants considered GH treatment for genetic and constitutional SS, respectively, 65% of them did not consider GH treatment for children with SS that was not caused by GH deficiency [15].

On the contrary, Alharbi et al. performed a cross-sectional study (n= 469 participants) among the families in the Qassim Region cities, including Buraydah, Unaizah, Al Rass, Riyadh Al Khabra, and outside the central region between 2021 and 2022. They highlighted the poor knowledge (68.4%) regarding the early detection and timely intervention of SS. However, being married, having children, or having a positive family history of SS were associated with higher knowledge levels [1]. Even worse, while several studies were performed on the prevalence of SS among children and adolescents [3,5,16,17], the literature is still scarce regarding the management of children with SS at the primary care level, especially in Saudi Arabia. Specifically, to our knowledge, no study has assessed the knowledge and attitude of primary health care (PHC) physicians in terms of the assessment and referral of patients with SS in Buraidah, Saudi Arabia.

The rationale for this study lies in the importance of early detection and timely intervention for children with SS. Early diagnosis and treatment initiation are crucial for a better prognosis, especially for pediatric candidates for GH treatment. Therefore, it is important to assess the management and referral practices of primary healthcare providers regarding patients with SS in Saudi Arabia. This study aims to identify the gaps and areas of improvement in awareness and practices of primary healthcare physicians in the region. By understanding their current knowledge, attitudes, barriers, and challenges, appropriate interventions and educational programs can be developed to improve the referral process, reduce unnecessary referrals, and enhance the quality of care provided to children with SS. Moreover, this study will contribute to the existing scarce literature on the knowledge and attitudes of primary healthcare providers regarding SS, particularly in the Saudi Arabian context.

Objectives

This study aimed to assess the current practices and barriers faced by primary healthcare physicians in the assessment and referral process for patients with SS. We also determined the awareness and attitude of the primary care physicians regarding the management of SS.

## Materials and methods

Study design and population

This study employed a cross-sectional design. The population of interest was primary healthcare physicians practicing in the Buraidah, Al-Qassim Region of Saudi Arabia. The inclusion criteria for this study include primary healthcare physicians practicing in the Buraidah, Al-Qassim Region of Saudi Arabia. Both genders and all nationalities were eligible for inclusion. However, rotating residents with a duration of less than 3 months and interns taking temporary rotations in PHC centers were excluded from the study. Additionally, physicians who were unwilling to participate or did not speak English were also excluded from the study.

Sampling and sample size

According to a recent study, there were a total of 144 primary health care (PHC) physicians in Buraidah [18]. We invited and included all PHC physicians in the study area who have been working for at least six months in the current PHC center. We excluded interns and ad-hoc primary care physicians. We used a consecutive sampling technique to include all available physicians who met the eligibility criteria.

Data sources and collection

The study utilized a validated questionnaire that has been previously used among PHC physicians in the Arabian Gulf countries [4]. The questionnaire was modified according to the local context. The questionnaire is specifically designed to assess the awareness and management practices of primary healthcare physicians regarding the assessment and referral of patients with SS. It includes general knowledge questions about short-stature definition, possible reasons for high percentages of referrals with limited clinical assessment, and management practices. The questionnaire was built and distributed on an electronic platform to facilitate obtaining responses and to enhance data management, while giving the participants the appropriate timeframe to complete and submit their responses.

Data management and analysis

Data was stored securely, and confidentiality was maintained throughout the study. Data entry was done using Microsoft Excel, and analysis was performed using Statistical Package for the Social Sciences (SPSS) version 30.0. Descriptive statistics were used to summarize the knowledge and attitudes of primary healthcare physicians. Independent sample t-tests were used to examine associations between socio-demographic characteristics and knowledge scores. Statistical significance was set at p < 0.05.

Ethical considerations

Ethical approval was obtained from the Qassim Regional Bioethics Committee (approval number: 607/46/3012). Informed consent was obtained from all study participants before their participation. Participation was voluntary, and participants had the right to withdraw from the study at any time without any consequences. Furthermore, the data was anonymous without the use of personal identifiers.

## Results

Demographics and professional information

This research involved 143 PHC physicians. The average age of the participants was 30.1 years (SD = 5.7). The majority were male, 75 (52.4%), and 68 (47.6%) were female. Most participants were Saudi nationals, 116 (81.1%), with the remaining 27 (18.9%) being non-Saudi. Regarding professional roles, 116 (81.1%) were family physicians, and 27 (18.9%) were general practitioners. A multidisciplinary team environment was reported by 83 (58%), while 60 (42%) worked independently. In terms of educational qualifications, about 54 (38%) had board-level qualifications, followed by MBBS 46 (32.2%). The average professional experience was 4.3 years (SD = 4.55), with a median of 3 years (IQR = 2.0 - 5.0 years), indicating a slightly skewed distribution toward fewer years of experience (Table 1).

**Table 1:** Socio-demographic and professional characteristics of participants (n = 143)

Variable	%(n)
Age	
Mean (SD)	30.1 (5.7)
Gender	
Male	52.4 (75)
Female	47.6 (68)
Nationality	
Saudi	81.1 (116)
Non-Saudi	18.9 (27)
What Is Your Specialty?	
General Practitioner	18.9 (27)
Family physicians	81.1 (116)
Work alone or are you part of a multidisciplinary team?	
Solo practitioner	42 (60)
Multidisciplinary team	58 (83)
Highest Qualification?	
MBBS	32.2 (46)
Diploma/Master	9.1 (13)
Board	37.8 (54)
Trainee	18.9 (27)
Experience	
Mean (SD)	4.3 (4.55)
Median (IQR)	3.0 (2.0 – 5.0)
Is growth hormone treatment covered in your practice by the government?	
Always	57.3 (82)
Sometimes	12.6 (18)
Never	11.9 (17)
I don’t know	18.2 (26)
Is growth hormone treatment covered in your practice by private insurance?	
Always	21 (30)
Sometimes	22.4 (32)
Never	16.8 (24)
I don't know	39.9 (57)
Which of the following would you like to hear/learn about (Yes)	
The causes and etiologies of children with short stature	88.1 (126)
Diagnosis of children with short stature	79 (113)
Management of short stature with growth hormone treatment	82.5 (118)
Safety of growth hormone treatment	98.6 (141)
How would you like to receive the learning about the above-selected topics? (Yes)	
Emails	26.6 (38)
Virtual meetings	64.3 (92)
Face-to-face meetings	51.7 (74)
Printed materials	39.2 (56)
Pharmaceutical representatives	25.9 (37)
Other	6.3 (9)

A majority, 126 (88.1%), expressed a desire to learn more about the causes and etiologies of SS, while 118 (82.5%) were interested in GH management. Additionally, 141 (98.6%) were keen to understand GH treatment safety, reinforcing previous findings on uncertainty regarding its risks. Preferred training methods varied, with 92 (64.3%) favoring virtual meetings, 74 (51.7%) opting for in-person sessions, 56 (39.2%) preferring printed materials, and 38 (26.6%) choosing email-based learning. 

Table 2 presents the understanding of SS among participants. A majority, 81 (56.6%), correctly defined SS as height below -2 standard deviations for age and gender. Regarding referral timing, 99 (69.2%) believed children should be referred when their height falls below the 5th percentile, aligning with clinical guidelines for early intervention. Knowledge about growth completion was relatively well understood, with 83 (58%) recognizing that growth ceases after puberty in females and 81 (56.6%) in males (Table 2).

**Table 2:** Primary health care physicians' awareness about short stature and referral processes (n = 143)

Variable	%(n)
Definition of short stature	
Height ≤1st percentile	2.8(4)
Height ≤3rd percentile	18.2(26)
Height ≤5th percentile	21.7(31)
Height 2 standard deviation below the mean adjusted for age and gender	56.6(81)
Age to complete growth for females	
15 years	7.0(10)
16 years	6.3(6.3)
17 years	0.7(1)
18 years	28(40)
Depends on the completion of puberty	58(83)
Age to complete growth for males	
15 years	4.2 (6)
16 years	2.1 (3)
17 years	1.4 (2)
18 years	35.7 (51)
Depends on the completion of puberty	56.6 (81)
Ideal age to refer a child with short stature	
<2 years	2.1 (3)
2–5 years	9.1(13)
5–10 years	16.1(23)
After the onset of puberty	3.5 (5)
As soon as height drops below the 5th percentile	69.2 (99)
Following conditions would you refer for growth hormone treatments (Yes)	
Down syndrome	21(30)
Silver–Russell syndrome	18.9(27)
Noonan syndrome	44.8(64)
Chronic renal failure	49.7(71)
Small for gestational age	49(70)
Prader-Willi syndrome	25.2(36)
Turner syndrome	26.6(38)
Growth hormone deficiencies	92.3(132)
Idiopathic short stature	81.1(117)

Doctors demonstrated varied understanding of GH therapy indications. While 132 (92.3%) correctly identified GH deficiency as a treatment indication, only 117 (81.1%) recognized idiopathic SS as a valid reason. Awareness of other GH-responsive conditions was notably lower, with only 38 (26.6%) identifying Turner syndrome, 25.2% recognizing Prader-Willi syndrome, 71 (49.7%) acknowledging chronic renal failure, and 64 (44.8%) recognizing Noonan syndrome.

The perception of GH therapy among doctors was mixed. While 79 (55.2%) considered it a viable option for SS, a significant proportion, 45 (31.5%), were uncertain, suggesting the need for further education. Similarly, only 75 (52.4%) believed GH treatment was safe, with 39 (27.3%) uncertain and 22 (15.4%) unsure. The hesitation around GH safety underscores the importance of addressing concerns about potential side effects and long-term impacts. Additionally, 80 (55.9%) believed caregivers favored GH treatment, whereas 58 (40.6%) thought caregivers were hesitant (Table 3).

**Table 3:** Attitudes of primary healthcare physicians toward growth hormone treatment (n = 143)

Variable	%(n)
Consider growth hormone treatment as an option for children with short stature?	
Yes	55.2 (79)
No	4.2 (6)
Maybe	31.5 (45)
I don’t know	9.1 (13)
Caregiver’s general reaction toward initiating growth hormone treatment for short stature?	
In Favor	55.9 (80)
Hesitant	40.6 (58)
Against	3.5 (5)
Do you consider growth hormone treatment for short stature safe?	
Yes	52.4 (75)
No	4.9 (7)
Maybe	27.3 (39)
I don’t know	15.4 (22)

Variability was observed in clinical practices related to short-stature evaluation. While 60 (42%) of doctors considered 18 years as the pediatric age limit, 45 (31.5%) used 14 years as the cutoff, indicating inconsistency in practice standards. Regarding patient encounters, 79 (55.2%) reported seeing 1-5 short-stature patients per month, whereas 61 (42.7%) saw none, reflecting differences in exposure to such cases. Growth assessment tools also varied. The majority, 103 (72%), used locally generated national growth charts, whereas 24 (16.8%) relied on WHO charts and 9 (6.3%) on CDC charts. Measurement methods were similarly diverse, with 67 (46.9%) using both wall-mounted scales and weight scales with extended height arms, while 28 (19.6%) relied solely on wall-mounted scales (Table 4).

**Table 4:** Practices of primary healthcare physicians about short stature (n = 143) CBC: complete blood count; IGF-1:insulin-like growth factor-1; IGFBP-3: insulin-like growth factor binding protein 3

Variable	%(n)
In your clinical practice, what is the cut-off age for pediatric patients?	
18th birthday	42.0 (60)
16th birthday	3.5 (5)
15th birthday	2.1 (3)
14th birthday	21 (30)
Younger than 14th birthday	31.5 (45)
How frequently do you see patients with short stature each month (new and follow-up)?	
1–5 patients	42.7 (61)
6–10 patients	55.2 (79)
10+ patients	2.1 (3)
Which of the following growth charts do you use most regularly?	
Locally generated (national) growth charts	72 (103)
CDC growth charts	6.3 (9)
WHO growth charts	16.8 (24)
I am not sure	4.9 (7)
How do you measure your patient’s height during the visits?	
Wall-mounted scale	19.6 (28)
Weight scale with an extended height-measuring arm	17.5 (25)
Both	46.9 (67)
None. I do not measure height	16.1 (23)
Which test(s) do you commonly order before referring the patient? (Yes)	
CBC	76.9 (110)
Thyroid function test	76.2 (109)
IGF-1	32.2 (46)
IGFBP-3	12.6 (18)
Electrolytes panel	36.4 (52)
Urine analysis	28 (40)
Liver enzymes	36.4 (52)
Celiac	34.3 (49)
Bone age	40.6 (58)
Karyotype	18.2 (26)
Growth hormone stimulation test	26.6 (38)
Brain MRI	7.7 (11)
Other	4.2 (6)
N/A	20.3 (29)
I do not order	21.7 (31)

Laboratory investigations before referral varied significantly. The most commonly requested tests included CBC 110 (76.9%) and thyroid function tests 109 (76.2%), reflecting standard initial screening. Bone age X-ray was ordered by 58 (40.6%), indicating that nearly half the doctors relied on radiological assessment. However, endocrine-specific tests were less frequently used; IGF-1 was ordered by only 46 (32.2%), GH stimulation testing by 38 (26.6%), and karyotyping by 26 (18.2%).

Analysis of knowledge scores revealed that non-Saudi physicians had a significantly higher average score (7.8) compared to their Saudi counterparts (6.8) (p = 0.033). However, no significant association was found between knowledge scores and other factors such as age, gender, specialty, work setting, or years of experience (Table 5).

**Table 5:** Comparison of knowledge of participants about short stature (n = 143) *statistically significant difference with a p-value of < 0.05.

Variable	Knowledge Score	P-value	
			Age				
Correlation coefficient	0.108	0.197		
Gender				
Male	6.9 (2.43)			
Female	7.2 (2.52)	0.468		
Nationality				
Saudi	6.8 (2.56)			
Non-Saudi	7.8 (1.89)	0.033*		
What is your specialty?				
General Practitioner	6.8 (2.73)	0.662		
Family physicians	7.1 (2.41)			
Work alone or are you part of a multidisciplinary team?				
solo practitioner	7.0 (2.40)			
multidisciplinary team	7.13 (2.53)	0.753		
Highest qualification?				
MBBS	6.6 (2.50)			
Diploma/Master	8.2 (1.92)			
Board	6.9 (2.51)			
Trainee	7.4 (2.577)	0.196		
Experience				
Correlation coefficient	0.128	0.128		

## Discussion

In our study, we found that 81 (56.6%) of the physicians correctly identified the cutoff of SS. In a study among physicians in the Arabian Gulf, similar were the findings, where 54.4% of the physicians identified height <2SD as a cutoff for SS [4]. This finding indicates that a large proportion of primary care physicians do not know the definition of SS. This requires that there should be training programs for physicians regarding SS. In the current study, 99 (69%) of the physicians would refer the child for further evaluation and management if the height falls below 5th percentile which is higher than the study by Kaplan et al. reported that 40.8% would refer as soon as the child’s height falls below 5th percentile [4]. Although in our sample a relatively higher proportion was referring timely, yet 44 (31%) still delayed the referral, which could affect the prognosis for the patients. Therefore, there should be clear guidelines for the referral of patients with SS.

In terms of treatment, 132 (92.3%) of the physicians correctly recognized GH deficiency as a valid indication for GH therapy-consistent with earlier studies conducted in Saudi Arabia [15]. However, fewer participants were aware of other approved indications: only about one-fourth identified Turner or Prader-Willi syndromes, and fewer than half recognized Noonan syndrome. These results echo regional findings and reveal an educational gap regarding the full scope of GH-responsive conditions [4,13]. Enhancing physician training on this topic could expand access to treatment for eligible children.

The attitudes toward GH treatment were cautious, with just over half of physicians supporting its use or safety. A considerable number expressed uncertainty-likely influenced by concerns about side effects, treatment costs, and long-term outcomes. Similar attitudes were noted in international studies, where physicians’ decisions were shaped by both medical guidelines and family expectations [13,14]. These findings reinforce the need to address misconceptions and provide physicians with up-to-date, evidence-based information.

When examining clinical practices, considerable variation was noted in growth measurement techniques. Only 67 (46.9%) of physicians used both wall-mounted and extended-arm measurement tools, despite the importance of accurate height assessment in diagnosis. Past research similarly noted limited use of standard equipment such as wall-mounted stadiometers [4]. Moreover, although most participants used national growth charts, a small number relied on international ones, which may not reflect local growth patterns. Encouraging standardized tools and methods could enhance diagnostic reliability.

In terms of laboratory workup, basic screening tests like CBC and thyroid function were commonly used. However, more specific endocrine assessments, such as IGF-1, GH stimulation, and karyotyping, were less frequently ordered, possibly due to their sequential nature or unavailability. This underuse mirrors findings by Hardin et al., who emphasized that such tests are essential for diagnosing endocrine causes of SS [13]. Furthermore, the availability of these tests in laboratories, referral procedures, follow-up, and physicians' knowledge about indications of these investigations may pose a barrier. This requires strengthened primary care lab capacity, effective linkage with hospital labs, and follow-up mechanisms. Furthermore, training programs should emphasize the role of comprehensive pre-referral evaluations.

Interestingly, non-Saudi physicians had higher knowledge scores compared to Saudi physicians, suggesting that prior training or exposure to different medical systems may influence familiarity with SS. Another possible reason could be the fact that, sub-sample of Saudi physicians might include junior doctors, while non-Saudi are more likely to have postgraduate qualifications. No significant differences were observed based on other factors like age, gender, or years of experience, indicating that continuous professional development may be more impactful than demographic traits.

Encouragingly, a majority of participants expressed strong interest in further training on SS and GH therapy. Preferences varied across virtual sessions, printed materials, and workshops, suggesting that blended learning strategies may be most effective for ongoing education.

This study is one of the few studies in the region to address the issue of physicians’ perspectives about SS. There are certain limitations that should be considered while interpreting the results of this study. Firstly, the sample was obtained from the Qassim region, which may limit the generalizability of the results to other settings. Secondly, there is a possibility of reporting bias, especially in those related to attitudes and practices. However, we assume this to have limited effects on the findings of our study, as we did an anonymized survey.

## Conclusions

The study points out significant knowledge gaps and differences in attitudes and practices regarding the evaluation and referral of children with SS among primary healthcare doctors. While most participants identified GH deficiency as a treatment indication, many were unsure about other eligible conditions, GH safety, and the appropriate referral process. These findings stress the need for focused educational programs, with an emphasis on GH therapy guidelines, diagnosis, and short-stature management. Addressing these gaps through structured training and improved guidelines could improve the quality of care and referral practices for children with SS in the region.

## Appendices

Questionnaire

Survey Section 1: Demographics

1.   Nationality?

a.   Saudi

b.   Non-Saudi

2.   Gender?

a.   Female

b.   Male

3.   Age? -------

4.   What is your specialty?

a.   General Practitioner

b.   Family physicians

5.   How many years have you been practicing medicine? -------years

6.   Do you work alone or are you part of a multidisciplinary team?

a.   I am a solo practitioner

b.   I work as part of a multidisciplinary team

Survey section 2: Short stature

7.   In your clinical practice, what is the cut off age for pediatric patients?

a.   18th birthday

b.   16th birthday

c.    15th birthday

d.   14th birthday

e.   Younger than 14th birthday

8.   How frequently do you see patients with short stature each month (new and follow-up)?

a.   None

b.   1-5 patients

c.    6-10 patients

d.   10+ patients

9.   What definition do you use for short stature?

a.   Height ≤1st percentile

b.   Height ≤3rd percentile

c.    Height ≤5th percentile

d.   Height <-2 standard deviations below the mean for matched gender and age

e.   Other

10.   Which of the following growth charts do you use most regularly?

a.   Locally generated (national) growth charts

b.   CDC growth charts

c.    WHO growth charts

d.   UK Royal College of Paediatrics and Child Health growth charts

e.   Other

f.     I am not sure

11.   How do you measure your patient's height during the visits?

a.   Wall-mounted scale

b.   Weight scale with an extended height-measuring arm

c.    Both

d.   None. I do not measure height

12.  At what age do you assume males/females complete their growth? (*please select one answer for each gender*)

a.   15 years

b.   16 years

c.    17 years

d.   18 years

e.   Depends on the completion of puberty

13.  As you responded 'yes' to the previous question, which test(s) do you commonly order before referring the patient? (*please select all that apply*)

a.   CBC

b.   Thyroid function test

c.    IGF-1

d.   IGFBP-3

e.   Electrolytes panel

f.     Urine analysis

g.   Liver enzymes

h.   Celiac

i.      Bone age

j.      Karyotype

k.    Growth Hormone stimulation test

l.      Brain MRI

m. Other

n.   N/A

14.  What is the ideal age to refer a child with short stature to a pediatric endocrinologist?

a.   <2 years

b.   2-5 years

c.    5-10 years

d.   After the onset of puberty

e.   As soon as height drops below 5th percentile

15. Do you consider growth hormone treatment an option for children with short stature?

a.   Yes

b.   No

c.    Maybe

d.   I don't know

16.  What is the caregiver's general reaction towards initiating growth hormone treatment for short stature?

a.   In favor

b.   Hesitant

c.    Against

17.   Do you consider growth hormone treatment for short stature safe?

a.   Yes

b.   No

c.    Maybe

d.   I don't know

18.  Which of the following conditions would you refer for growth hormone treatments? (*please select all that apply*)

a.   Down syndrome

b.   Silver-Russell syndrome

c.    Noonan syndrome

d.   Chronic renal failure

e.   Small for gestational age

f.     Prader-Willi syndrome

g.   Turner syndrome

h.   Growth Hormone deficiencies

i.      Idiopathic short stature

19.  Is growth hormone treatment covered in your practice? *(please select one answer for government and one answer for insurance)*

Government:

a.   Always

b.   Sometimes

c.    Never

d.   I don't know

Insurance:

a.   Always

b.   Sometimes

c.    Never

d.   I don't know

20.   Which of the following would you like to hear/learn about *(please select all that apply)*

a.   The causes and etiologies of children with short stature

b.   Diagnosis of children with short stature

c.    Management of short stature with growth hormone treatment

d.   Safety of growth hormone treatment

21.  How would you like to receive the learning about the above selected topics?

a.   Emails

b.   Virtual meetings

c.    Face-to-face meetings

d.   Printed materials

e.   Pharmaceutical representatives

f.     Other

## References

[1] Alharbi MS, Alatni RI, Alhammad RA (2022). Knowledge and awareness for the early detection and intervention of short stature among families in Qassim region 2021-2022: A cross-sectional study. Open Access Maced J Med Sci.

[2] Polidori N, Castorani V, Mohn A, Chiarelli F (2020). Deciphering short stature in children. Ann Pediatr Endocrinol Metab.

[3] El-Shafie AM, Kasemy ZA, Omar ZA (2020). Prevalence of short stature and malnutrition among Egyptian primary school children and their coexistence with Anemia. Ital J Pediatr.

[4] Kaplan W, Al Amiri E, Attia N (2022). Assessment and referral of patients with short stature by primary care physicians in the Arabian gulf region: Current perspectives from a regional survey. Front Pediatr.

[5] Velayutham K, Selvan SS, Jeyabalaji RV, Balaji S (2017). Prevalence and etiological profile of short stature among school children in a South Indian population. Indian J Endocrinol Metab.

[6] Voss LD, Mulligan J, Betts PR, Wilkin TJ (1992). Poor growth in school entrants as an index of organic disease: The Wessex growth study. BMJ.

[7] Al-Jurayyan N NA, Mohamed SH, Al Otaibi HM, Al Issa ST, Omer HG (2012). Short stature in children: Pattern and frequency in a pediatric clinic, Riyadh, Saudi Arabia. Sudan J Paediatr.

[8] El Mouzan MI, Al Herbish AS, Al Salloum AA, Al Omer AA, Qurachi MM (2012). Regional prevalence of short stature in Saudi school-age children and adolescents. ScientificWorldJournal.

[9] Maghnie M, Labarta JI, Koledova E, Rohrer TR (2017). Short stature diagnosis and referral. Front Endocrinol.

[10] Rani D, Shrestha R, Kanchan T, Krishan K (2025). Short Stature. StatPearls.

[11] Kamoun C, Largent EA, Grimberg A (2024). Heightism, growth hormone treatment, and social functioning: A holistic approach to a persistent clinical challenge. Curr Opin Pediatr.

[12] Silvers JB, Marinova D, Mercer MB, Connors A, Cuttler L (2010). A national study of physician recommendations to initiate and discontinue growth hormone for short stature. Pediatrics.

[13] Hardin DS, Woo J, Butsch R, Huett B (2007). Current prescribing practices and opinions about growth hormone therapy: Results of a nationwide survey of paediatric endocrinologists. Clin Endocrinol.

[14] Al Herbish AS, Al Alwan I, Al Mutair A (2014). Growth hormone therapy and treatment outcomes: Current clinical practice of the Gulf Cooperation Council. Expert Rev Endocrinol Metab.

[15] Al-Herbish AS, Al-Jurayyan NA, Abobakr AM, Al-Nuaim AA (2000). Growth hormone: Do we have a national perspective of indications for its use?. Saudi Med J.

[16] Al-Abdulrazzaq D, Al-Taiar A, Hassan K, Al-Twari B, Al-Osaimi A, Al-Busairi I (2016). Referral pattern of children with short stature to a pediatric endocrine clinic in Kuwait. J Pediatr Endocrinol Metab.

[17] Zayed AA, Beano AM, Haddadin FI (2016). Prevalence of short stature, underweight, overweight, and obesity among school children in Jordan. BMC Public Health.

[18] Almatrudi TA, Rabbani U (2022). Awareness, attitude, and practice of evidence-based medicine among primary healthcare physicians in Buraidah, Saudi Arabia. J Family Med Prim Care.

